# Poly[chlorido[μ_4_-2,2′-(2-methyl-1*H*-benzimidazol-3-ium-1,3-di­yl)diacetato]­zinc]

**DOI:** 10.1107/S1600536812020077

**Published:** 2012-05-12

**Authors:** Jia-Qin Liu, Zhen-Jü Jiang, Zhi-Hong Xu, Yan Zhang

**Affiliations:** a School of Physics and Chemistry, Xihua University, Chengdu 610039, People’s Republic of China

## Abstract

The title compound, [Zn(C_12_H_11_N_2_O_4_)Cl]_*n*_, contains a centrosymmetric dimetal tetra­carboxyl­ate paddle-wheel moiety in which the Zn^II^ atom is square-pyramidally coordinated by four carboxyl­ate O atoms at the basal positions and one Cl^−^ anion at the apical position. Each paddle-wheel unit is joined to four such neighbours through bridging dicarboxyl­ate ligands, producing a two-dimensional undulating layer parallel to (-101). Adjacent sheets are stacked in a parallel fashion to form a three-dimensional supra­molecular structure which is stabilized by inter­layer π–π inter­actions between benzene rings, with a centroid–centroid distance of 3.722 Å. The range of Zn—O bond lengths is 2.0440 (17)–2.1256 (15) Å and the Zn—Cl bond length is 2.2622 (6) Å.

## Related literature

For background to and potential applications of carboxyl­ate-containing coordination polymers, see Bourne *et al.* (2001 ▶); Chen *et al.* (2005 ▶); Kitagawa *et al.* (2004 ▶); Li *et al.* (2012 ▶); Xuan *et al.* (2012 ▶).
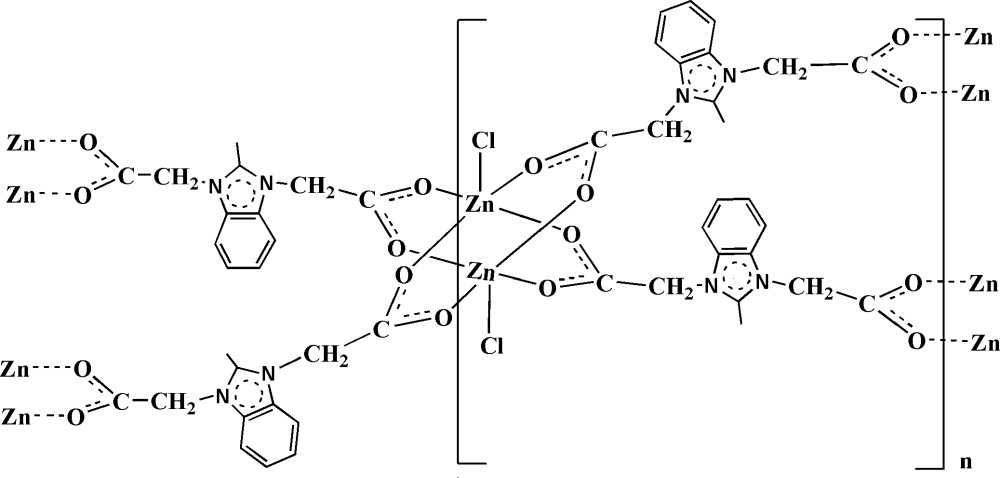


## Experimental

### 

#### Crystal data

[Zn(C_12_H_11_N_2_O_4_)Cl]
*M* *_r_* = 348.05
Monoclinic, 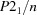

*a* = 7.1285 (17) Å
*b* = 13.301 (3) Å
*c* = 12.804 (3) Å
β = 90.540 (4)°
*V* = 1214.0 (5) Å^3^
*Z* = 4
Mo *K*α radiation
μ = 2.26 mm^−1^
*T* = 173 K
0.48 × 0.32 × 0.30 mm

#### Data collection

Bruker SMART CCD area-detector diffractometer
Absorption correction: multi-scan (*SADABS*; Bruker, 1998 ▶) *T*_min_ = 0.424, *T*_max_ = 0.508
6072 measured reflections
2640 independent reflections
2327 reflections with *I* > 2σ(*I*)
*R*_int_ = 0.026

#### Refinement

*R*[*F* ^2^ > 2σ(*F* ^2^)] = 0.027
*wR*(*F* ^2^) = 0.078
*S* = 1.07
2640 reflections
181 parameters
H-atom parameters constrained
Δρ_max_ = 0.45 e Å^−3^
Δρ_min_ = −0.45 e Å^−3^

### 

Data collection: *SMART* (Bruker, 1998 ▶); cell refinement: *SAINT* (Bruker, 1998 ▶); data reduction: *SAINT*; program(s) used to solve structure: *SHELXS97* (Sheldrick, 2008 ▶); program(s) used to refine structure: *SHELXL97* (Sheldrick, 2008 ▶); molecular graphics: *SHELXTL* (Sheldrick, 2008 ▶); software used to prepare material for publication: *SHELXTL*.

## Supplementary Material

Crystal structure: contains datablock(s) I, global. DOI: 10.1107/S1600536812020077/bg2452sup1.cif

Structure factors: contains datablock(s) I. DOI: 10.1107/S1600536812020077/bg2452Isup2.hkl

Additional supplementary materials:  crystallographic information; 3D view; checkCIF report

## Figures and Tables

**Fig. 1.**
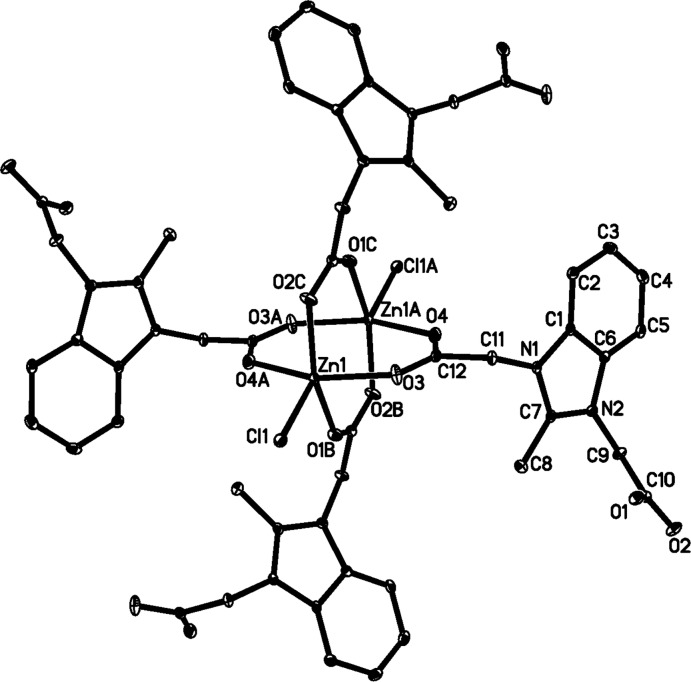
ORTEP drawing (at 30% probability) of a paddle-wheel unit in the title compound (symmetry codes: A, -x, -y, -z; B, -0.5 + x, 1.5 - y, -0.5 + z; C, 0.5 - x, -0.5 + y, 1.5 - z). Hydrogen atoms are omitted for clarity.

**Fig. 2.**
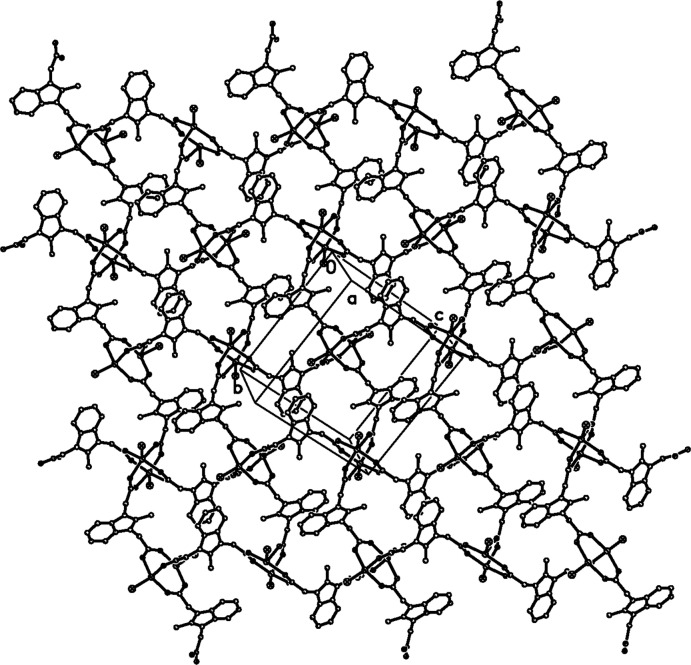
The 2-D sheet structure parallel to the (-101) plane, hydrogen atoms are omitted for clarity.

## supplementary crystallographic information

### Comment

Carboxylate-containing ligands have been intensively investigated to construct metal-organic frameworks with an intriguing variety of topologies and potential applications in gas sorption, separation and/or catalysis (Bourne *et al.*, 2001; Chen *et al.*, 2005; Kitagawa *et al.*, 2004; Li *et al.*, 2012; Xuan *et al.*, 2012). Polycarboxylate ligands with suitable spacers are good choices for such architectures because the topological structures can be adjusted not only by carboxylate groups but also by the organic spacers. Here we use a flexible zwitterionic ligand, 1-acetoxy-2-methylbenzimidazole-3-acetate acid [HL], to prepare the title compound [Zn(*L*)Cl]_n_ (I).

The motive consists of a centrosymmetric paddle-wheel dimetal tetracarboxylate moiety [Zn_2_(CO_2_)_4_] (Fig. 1) in which each Zn^II^ is square-pyramidally coordinated by four carboxylate oxygen atoms at the basal position and one Cl^-^ anion at the apical position. Each paddle-wheel unit is bridged by four such neighbors through bridging dicarboxylate ligands, producing a two-dimensional undulate layer in which π-π interactions between phenyl rings of benzimidazole moieties (ring-centroid distance: 3.579 (2) Å) cooperate in the 2-D sheet formation (Fig. 2). Adjacent sheets are stacked in a parallel fashion to form a 3-D supramolecular structure stabilized by interlayer π-π interactions between phenyl rings with a ring-centroid distance of 3.722 (2) Å. The Zn—O span is 2.0440 (17)-2.1256 (15) Å and the Zn—Cl distance is 2.2622 (6) Å.

### Experimental

After the pH of an ethanol/water mixture solution (10 ml with ratio of 4:1) containing ZnCl_2_.2H_2_O (0.0408 g, 0.3 mmol) and the HL ligand (0.0498 g,0.2 mmol) was adjusted to 7 by addition of triethylamine, the resulting solution was sealed in a Teflon-lined steel bomb (25 ml) and then heated at 140°C for 2 days. Colorless block crystals were collected. Yield: 16%. Elemental analysis (%) calcd for the title compound: C 41.38, H 3.16, N 8.04; found: C 41.24, H 3.23, N 8.47. IR: 1672(*s*), 1472(*m*), 1436(*m*), 1389(*s*), 1310(*m*), 764(*s*), 721(*m*), 619(*m*), 574(*m*).

### Refinement

All hydrogen atoms were generated geometrically and refined with a riding model, *U*_iso_(H)= x×*U*_eq_(Host) (aromatic: C—H: 0.95Å, x=1.2; methyl, C—H: 0.98Å, x=1.5; methylene, C—H: 0.99Å, x=1.2)

### Crystal data

**Table:**

[Zn(C_12_H_11_N_2_O_4_)Cl]	*Z* = 4
*M**_r_* = 348.05	*F*(000) = 704
Monoclinic, *P*2_1_/*n*	*D*_x_ = 1.904 Mg m^−^^3^
Hall symbol: -P 2yn	Mo *K*α radiation, λ = 0.71073 Å
*a* = 7.1285 (17) Å	θ = 2.2–27.1°
*b* = 13.301 (3) Å	µ = 2.26 mm^−^^1^
*c* = 12.804 (3) Å	*T* = 173 K
β = 90.540 (4)°	Block, colorless
*V* = 1214.0 (5) Å^3^	0.48 × 0.32 × 0.30 mm

### Data collection

**Table:**

Bruker SMART CCD area-detector diffractometer	2640 independent reflections
Radiation source: fine-focus sealed tube	2327 reflections with *I* > 2σ(*I*)
Graphite monochromator	*R*_int_ = 0.026
ω scans	θ_max_ = 27.1°, θ_min_ = 2.2°
Absorption correction: multi-scan (*SADABS*; Bruker, 1998)	*h* = −9→8
*T*_min_ = 0.424, *T*_max_ = 0.508	*k* = −11→17
6072 measured reflections	*l* = −16→9

### Refinement

**Table:**

Refinement on *F*^2^	Primary atom site location: structure-invariant direct methods
Least-squares matrix: full	Secondary atom site location: difference Fourier map
*R*[*F*^2^ > 2σ(*F*^2^)] = 0.027	Hydrogen site location: inferred from neighbouring sites
*wR*(*F*^2^) = 0.078	H-atom parameters constrained
*S* = 1.07	*w* = 1/[σ^2^(*F*_o_^2^) + (0.0438*P*)^2^ + 0.6356*P*] where *P* = (*F*_o_^2^ + 2*F*_c_^2^)/3
2640 reflections	(Δ/σ)_max_ = 0.001
181 parameters	Δρ_max_ = 0.45 e Å^−^^3^
0 restraints	Δρ_min_ = −0.45 e Å^−^^3^

### Special details

**Table:**

Geometry. All e.s.d.'s (except the e.s.d. in the dihedral angle between two l.s. planes) are estimated using the full covariance matrix. The cell e.s.d.'s are taken into account individually in the estimation of e.s.d.'s in distances, angles and torsion angles; correlations between e.s.d.'s in cell parameters are only used when they are defined by crystal symmetry. An approximate (isotropic) treatment of cell e.s.d.'s is used for estimating e.s.d.'s involving l.s. planes.
Refinement. Refinement of *F*^2^ against ALL reflections. The weighted *R*-factor *wR* and goodness of fit *S* are based on *F*^2^, conventional *R*-factors *R* are based on *F*, with *F* set to zero for negative *F*^2^. The threshold expression of *F*^2^ > σ(*F*^2^) is used only for calculating *R*-factors(gt) *etc*. and is not relevant to the choice of reflections for refinement. *R*-factors based on *F*^2^ are statistically about twice as large as those based on *F*, and *R*- factors based on ALL data will be even larger.

### Fractional atomic coordinates and isotropic or equivalent isotropic displacement parameters (Å^2^)

**Table:**

	*x*	*y*	*z*	*U*_iso_*/*U*_eq_	
Zn1	0.13104 (3)	0.553340 (17)	0.417142 (17)	0.01203 (10)	
Cl1	0.29440 (7)	0.65999 (4)	0.31470 (4)	0.01697 (13)	
O1	0.4360 (2)	0.84000 (11)	0.97522 (12)	0.0181 (3)	
O2	0.2502 (2)	0.91230 (12)	1.09352 (13)	0.0217 (4)	
O3	0.2669 (2)	0.56544 (13)	0.55787 (12)	0.0241 (4)	
O4	0.0830 (2)	0.49535 (12)	0.67900 (12)	0.0203 (3)	
N1	0.3143 (2)	0.58092 (13)	0.83280 (13)	0.0122 (3)	
N2	0.2081 (2)	0.67202 (13)	0.96116 (14)	0.0131 (4)	
C1	0.2938 (3)	0.51606 (15)	0.91696 (16)	0.0123 (4)	
C2	0.3314 (3)	0.41392 (16)	0.92770 (17)	0.0164 (4)	
H2A	0.3829	0.3755	0.8724	0.020*	
C3	0.2899 (3)	0.37134 (16)	1.02313 (18)	0.0190 (5)	
H3A	0.3135	0.3018	1.0338	0.023*	
C4	0.2140 (3)	0.42806 (17)	1.10429 (18)	0.0190 (5)	
H4A	0.1854	0.3957	1.1683	0.023*	
C5	0.1792 (3)	0.53041 (16)	1.09412 (16)	0.0157 (4)	
H5A	0.1280	0.5689	1.1495	0.019*	
C6	0.2235 (3)	0.57346 (15)	0.99858 (16)	0.0133 (4)	
C7	0.2610 (3)	0.67345 (15)	0.86146 (16)	0.0128 (4)	
C8	0.2578 (3)	0.75932 (16)	0.78819 (17)	0.0197 (5)	
H8A	0.2142	0.8196	0.8246	0.030*	
H8B	0.1726	0.7443	0.7297	0.030*	
H8C	0.3844	0.7709	0.7617	0.030*	
C9	0.1487 (3)	0.75902 (15)	1.02331 (17)	0.0159 (4)	
H9A	0.1187	0.7357	1.0946	0.019*	
H9B	0.0321	0.7867	0.9920	0.019*	
C10	0.2944 (3)	0.84401 (15)	1.03157 (16)	0.0136 (4)	
C11	0.3852 (3)	0.55606 (16)	0.72945 (16)	0.0142 (4)	
H11A	0.4637	0.4948	0.7348	0.017*	
H11B	0.4662	0.6116	0.7051	0.017*	
C12	0.2295 (3)	0.53811 (15)	0.64823 (17)	0.0138 (4)	

### Atomic displacement parameters (Å^2^)

**Table:**

	*U*^11^	*U*^22^	*U*^33^	*U*^12^	*U*^13^	*U*^23^
Zn1	0.01287 (15)	0.01381 (14)	0.00939 (14)	−0.00070 (8)	−0.00114 (9)	0.00073 (8)
Cl1	0.0203 (3)	0.0164 (2)	0.0142 (2)	−0.00402 (19)	0.00246 (19)	−0.00035 (18)
O1	0.0171 (8)	0.0169 (7)	0.0205 (8)	−0.0037 (6)	0.0038 (6)	−0.0020 (6)
O2	0.0251 (9)	0.0166 (7)	0.0235 (8)	−0.0044 (6)	0.0047 (7)	−0.0089 (7)
O3	0.0191 (8)	0.0415 (10)	0.0114 (8)	−0.0005 (7)	−0.0032 (6)	0.0026 (7)
O4	0.0199 (8)	0.0227 (8)	0.0181 (8)	−0.0067 (7)	−0.0050 (6)	−0.0012 (6)
N1	0.0123 (8)	0.0143 (8)	0.0100 (8)	−0.0009 (7)	−0.0026 (6)	−0.0012 (7)
N2	0.0136 (9)	0.0126 (8)	0.0130 (8)	−0.0012 (7)	−0.0004 (7)	−0.0009 (7)
C1	0.0113 (10)	0.0153 (10)	0.0102 (9)	−0.0027 (8)	−0.0039 (7)	−0.0003 (8)
C2	0.0161 (10)	0.0153 (10)	0.0176 (10)	0.0004 (8)	−0.0048 (8)	−0.0025 (8)
C3	0.0172 (11)	0.0136 (10)	0.0261 (12)	−0.0031 (8)	−0.0065 (9)	0.0049 (9)
C4	0.0170 (11)	0.0222 (11)	0.0178 (11)	−0.0050 (9)	−0.0047 (8)	0.0066 (9)
C5	0.0153 (10)	0.0206 (10)	0.0112 (10)	−0.0045 (8)	−0.0019 (8)	−0.0014 (8)
C6	0.0117 (9)	0.0136 (9)	0.0145 (10)	−0.0035 (8)	−0.0032 (8)	−0.0012 (8)
C7	0.0110 (9)	0.0150 (10)	0.0121 (9)	−0.0027 (8)	−0.0038 (7)	−0.0015 (8)
C8	0.0264 (12)	0.0172 (10)	0.0154 (10)	0.0003 (9)	−0.0016 (9)	0.0030 (9)
C9	0.0154 (10)	0.0131 (10)	0.0191 (10)	−0.0007 (8)	0.0028 (8)	−0.0055 (8)
C10	0.0152 (10)	0.0131 (9)	0.0126 (9)	−0.0012 (8)	−0.0025 (8)	−0.0003 (8)
C11	0.0134 (10)	0.0198 (11)	0.0093 (9)	0.0010 (8)	−0.0014 (8)	−0.0014 (8)
C12	0.0142 (10)	0.0150 (10)	0.0121 (9)	0.0045 (8)	−0.0034 (8)	−0.0034 (8)

### Geometric parameters (Å, º)

**Table:**

Zn1—O3	2.0440 (17)	C2—H2A	0.9500
Zn1—O4^i^	2.0559 (16)	C3—C4	1.397 (3)
Zn1—O2^ii^	2.0632 (16)	C3—H3A	0.9500
Zn1—O1^iii^	2.1256 (15)	C4—C5	1.390 (3)
Zn1—Cl1	2.2622 (6)	C4—H4A	0.9500
O1—C10	1.247 (3)	C5—C6	1.390 (3)
O2—C10	1.248 (3)	C5—H5A	0.9500
O3—C12	1.244 (3)	C7—C8	1.478 (3)
O4—C12	1.256 (3)	C8—H8A	0.9800
N1—C7	1.340 (3)	C8—H8B	0.9800
N1—C1	1.389 (3)	C8—H8C	0.9800
N1—C11	1.459 (3)	C9—C10	1.538 (3)
N2—C7	1.335 (3)	C9—H9A	0.9900
N2—C6	1.400 (3)	C9—H9B	0.9900
N2—C9	1.469 (3)	C11—C12	1.532 (3)
C1—C2	1.391 (3)	C11—H11A	0.9900
C1—C6	1.392 (3)	C11—H11B	0.9900
C2—C3	1.381 (3)		
			
O3—Zn1—O4^i^	153.56 (7)	C6—C5—H5A	121.8
O3—Zn1—O2^ii^	86.48 (7)	C4—C5—H5A	121.8
O4^i^—Zn1—O2^ii^	88.65 (7)	C5—C6—C1	121.4 (2)
O3—Zn1—O1^iii^	86.86 (7)	C5—C6—N2	132.0 (2)
O4^i^—Zn1—O1^iii^	86.31 (7)	C1—C6—N2	106.52 (18)
O2^ii^—Zn1—O1^iii^	154.17 (6)	N2—C7—N1	109.40 (18)
O3—Zn1—Cl1	102.68 (5)	N2—C7—C8	127.98 (19)
O4^i^—Zn1—Cl1	103.50 (5)	N1—C7—C8	122.58 (19)
O2^ii^—Zn1—Cl1	108.55 (5)	C7—C8—H8A	109.5
O1^iii^—Zn1—Cl1	97.25 (5)	C7—C8—H8B	109.5
C10—O1—Zn1^iv^	135.18 (14)	H8A—C8—H8B	109.5
C10—O2—Zn1^v^	120.91 (14)	C7—C8—H8C	109.5
C12—O3—Zn1	133.86 (15)	H8A—C8—H8C	109.5
C12—O4—Zn1^i^	124.71 (14)	H8B—C8—H8C	109.5
C7—N1—C1	109.00 (17)	N2—C9—C10	114.73 (17)
C7—N1—C11	123.92 (17)	N2—C9—H9A	108.6
C1—N1—C11	127.07 (18)	C10—C9—H9A	108.6
C7—N2—C6	108.60 (17)	N2—C9—H9B	108.6
C7—N2—C9	126.32 (18)	C10—C9—H9B	108.6
C6—N2—C9	125.06 (18)	H9A—C9—H9B	107.6
N1—C1—C2	131.4 (2)	O1—C10—O2	127.5 (2)
N1—C1—C6	106.45 (18)	O1—C10—C9	118.58 (18)
C2—C1—C6	122.14 (19)	O2—C10—C9	113.85 (18)
C3—C2—C1	116.4 (2)	N1—C11—C12	113.33 (17)
C3—C2—H2A	121.8	N1—C11—H11A	108.9
C1—C2—H2A	121.8	C12—C11—H11A	108.9
C2—C3—C4	121.6 (2)	N1—C11—H11B	108.9
C2—C3—H3A	119.2	C12—C11—H11B	108.9
C4—C3—H3A	119.2	H11A—C11—H11B	107.7
C5—C4—C3	122.0 (2)	O3—C12—O4	127.6 (2)
C5—C4—H4A	119.0	O3—C12—C11	115.17 (19)
C3—C4—H4A	119.0	O4—C12—C11	117.18 (19)
C6—C5—C4	116.4 (2)		

Symmetry codes: (i) −*x*, −*y*+1, −*z*+1; (ii) −*x*+1/2, *y*−1/2, −*z*+3/2; (iii) *x*−1/2, −*y*+3/2, *z*−1/2; (iv) *x*+1/2, −*y*+3/2, *z*+1/2; (v) −*x*+1/2, *y*+1/2, −*z*+3/2.
